# Temporary Treatment during Primary HIV Infection Does Not Affect Virologic Response to Subsequent Long-Term Treatment

**DOI:** 10.1371/journal.pone.0089639

**Published:** 2014-04-03

**Authors:** Marlous L. Grijsen, Ferdinand W. N. M. Wit, Suzanne Jurriaans, Frank P. Kroon, Emile F. Schippers, Peter Koopmans, Luuk Gras, Joep M. A. Lange, Jan M. Prins, on behalf of the Primo-SHM study group

**Affiliations:** 1 Academic Medical Center, University of Amsterdam, Department of Internal Medicine, Division of Infectious Diseases, Center for Infection and Immunity Amsterdam, Amsterdam, the Netherlands; 2 Department of Global Health, Amsterdam Institute for Global Health and Development, Amsterdam, the Netherlands; 3 Academic Medical Center, University of Amsterdam, Department of Medical Microbiology, Amsterdam, the Netherlands; 4 Leiden University Medical Center, Department of Infectious Diseases, Leiden, the Netherlands; 5 Haga Hospital, location Leyenburg, Department of Internal Medicine, The Hague, the Netherlands; 6 Radboud University Medical Center, Department of Internal Medicine, Nijmegen, the Netherlands; 7 HIV Monitoring Foundation, Amsterdam, the Netherlands; Centro de Biología Molecular Severo Ochoa (CSIC-UAM), Spain

## Abstract

Temporary cART during primary HIV-infection (PHI) did not select for drug resistance mutations after treatment interruption and did not affect the subsequent virological response to long-term cART. Our data demonstrate that fear of drug resistance development is not a valid argument to refrain from temporary early treatment during PHI.

## Introduction

In the Primo-SHM trial, a multicenter randomized trial comparing no treatment with 24- or 60-weeks of combination antiretroviral therapy (cART) during primary HIV-infection (PHI), we recently demonstrated that temporary early cART lowered the viral setpoint and deferred the need for reinitiation of cART during chronic HIV-infection [1]. Two other randomized studies also observed a modest delay in disease progression after a short course of cART in PHI [2], [3]. However, an important concern of temporary early cART, and of structured treatment interruptions (TI) in general, is the risk of developing drug resistance mutations after TI, especially in the case of NNRTI-based regimens, which compromise future treatment options.

The aim of this study was to assess the effect of temporary cART during PHI on the subsequent virologic response to long-term cART, in patients who previously participated in the Primo-SHM trial. To this end, we compared the viral decay and the time to viral (re)suppression between the early treated patients who reinitiated cART and the patients in whom treatment was deferred until conventional criteria to start long-term cART had been reached.

## Methods

Between May 2003 and March 2010 168 patients with laboratory evidence of PHI were randomized in the Primo-SHM trial to receive no treatment (naive patients, n = 36) or 24 or 60 weeks of early cART (early treated patients, n = 132) [1]. PHI was defined as a negative or indeterminate Western blot combined with a detectable plasma viral load (pVL), or, in case of a positive Western blot, a negative HIV-screening test result ≤180 days. Early cART consisted of a quadruple triple-class regimen containing two NRTIs (zidovudine/lamivudine 300/150 mg bid), an NNRTI (efavirenz 600 mg qd) and a boosted PI (lopinavir/ritonavir capsules 533/133 mg bid). The latter was discontinued when the pVL had dropped <50 copies/ml. After January 2008 zidovudine/lamivudine was replaced by tenofovir/emtricitabine (245/200 mg qd) and lopinavir/ritonavir tablets (600/150 mg bid) replaced the capsules. Changes to this regimen were allowed in case of transmitted drug resistance or if one of the drugs was not tolerated. The study protocol required patients to reach a pVL <50 copies/ml before interrupting therapy as scheduled. Long-term cART was (re)started in case of two consecutive CD4 cell counts below 350 cells/mm^3^, severe constitutional symptoms, the occurrence of an AIDS defining event, or if the patient preferred on (re)initiating cART. Follow-up visits after (re)start of cART were scheduled according to standard treatment guidelines, i.e. after four weeks of treatment and every three months thereafter. This study was approved by the Medical Ethics Committee of the Academic Medical Hospital in Amsterdam, the Netherlands, and written informed consent was obtained from all participants.

In the current study, we included the 94 out of 168 participants (56%) who had started or restarted long-term cART by September 2011 and who had at least one pVL measurement after (re)initiation of cART. (Re)Start regimens were at the discretion of the treating physician. Resistance testing was performed at diagnosis of PHI. To investigate possible acquired resistance during or after stopping of early cART, we performed additional resistance testing of the reverse transcriptase gene retrospectively in the first stored plasma sample obtained within one year after TI with a pVL above 3.0 log_10_ c/ml. Data of the 24- and 60-weeks early treated patients were combined in all analyses because the viral decay was not significantly different between the two groups (data not shown).

Sociodemographic characteristics and laboratory data at (re)start of long-term cART were compared between the naive and early treated patients using chi-square, Fisher's exact and Kruskal-Wallis tests where appropriate. Viral decay after start/restart of cART in naive/early treated patients was analysed using linear mixed models incorporating repeated measurements, which showed a tri-phasic pattern with distinct slopes from week zero to four, week four to eight and from week eight onwards. For this analysis patients were censored once they reached a pVL <50 c/ml. A similar analysis was done for the early treated group, comparing viral decay during early initial cART with the decay after subsequent restart of cART. Time to viral (re)suppression, defined as a pVL <50 c/ml, was compared between the two groups using Kaplan-Meier plots and multivariable Cox regression analysis. The proportion of patients having an undetectable viral load (<50 c/mL) and the mean (SD) CD4+ T cell count in the two groups were plotted and compared at 24-week intervals for 144 weeks after the (re-)start of cART. All analyses ignored modifications of treatment regimens, but censored patients at the moment of interruption of cART for more than two weeks. Data were analyzed using SAS version 9.2 (SAS institute, USA).

## Results

Of the 36 naive and 132 early treated participants in the Primo-SHM trial, 31 (86%) and 63 (48%) had (re)initiated long-term cART by September 2011, respectively, and were included in this study. At the time of diagnosis of PHI (so before any treatment was started) the pVL and CD4 count data of the naïve and early treated patients were 5.14 (SD 0.90) versus 5.34 (0.73) log_10_ c/ml (*P* = 0.24) and 446 (SD 164) versus 481 (236) cells/mm^3^ (*P* = 0.46), respectively. Additionally, the naïve patients had less transmitted drug resistance mutations as compared to the early treated patients at the moment of PHI-diagnosis (0 versus 8 (14%); *P* = 0.048). 5/8 early treated patients carried a M41L mutation, 2 patients a M46I mutation of whom one also had a T215S mutation and one carried a K103N mutation. In 52/63 early treated patients all antiretroviral drugs had been stopped simultaneously at TI: at that moment 31/63 (49%) were receiving dual-class NNRTI-based therapy, 15/63 (24%) dual-class boosted PI-based therapy, and 6/63 (10%) triple-class therapy. In the remaining 11/63 patients (17%) a staggered TI method was used, in which the NNRTI was stopped prior to the NRTI-backbone. Six early treated patients (6%) did not have a pVL <50 c/ml at TI (range 58–1882 copies/ml). The median time between TI and restart of long-term cART was 1.9 (IQR 0.9–3.1) years.

89/94 (re)starting participants (95%) were men. Mean age and CD4 count at (re)start of long-term cART were 44 (SD 9) years and 290 (110) cells/mm^3^, respectively, and were not significantly different between the naive and early treated patients. The naive patients had a higher mean pVL at (re)start (5.0 (SD 0.7) versus 4.7 (0.7) log_10_ c/ml; *P* = 0.07). Naive patients initiated long-term cART more often with an NNRTI-containing regimen than early treated patients (24 (77%) versus 37 (59%); *P* = 0.10). Four naive (13%) and 23 early treated patients (37%) (re)started long-term cART with a boosted PI (*P* = 0.03), and three (10%) versus three (5%) patients, respectively, (re)initiated with triple-class therapy (*P* = 0.40).

Drug resistance testing was performed after TI in 56/63 (89%) early treated participants. None of these patients had developed a novel (acquired) drug resistance mutation. Of the remaining seven patients, one harboured a 103N mutation, which was already present at diagnosis of PHI (this patient had started with a quadruple NNRTI-based regimen before resistance testing results were known, once results were available the regimen was adapted), and for six patients no stored samples were available or the first available stored sample was older than one year. The median interval between TI and the timepoint of resistance testing was 5.0 (IQR 4.0–8.0) weeks. In 3/56 patients the time point of resistance testing was >12 weeks after TI. Other patient characteristics (transmission route, ethnicity, history of CDC-events, virus subtype) were comparable between the two groups. One early treated patient was lost-to-follow-up after restart of long-term cART and he was censored at this visit. Patient characteristics are summarized in Table 1.

**Table 1:** Patient characteristics at (re)initiation of long-term cART.

	Total (N = 94)	No treatment during PHI (N = 31)	Early cART during PHI (N = 63)	*P*-value
**Age** (years), mean (SD)	44 (9)	44 (10)	43 (8)	0.9
**Men**	89 (95)	31 (100)	58 (92)	0.2
**MSM**	78 (83)	26 (84)	52 (83)	0.9
**Born in the Netherlands**	84 (89)	28 (90)	56 (89)	1.0
**History of CDC C-event**	11 (12)	2 (7)	9 (14)	0.3
**CD4 count** (cells/mm^3^), mean (SD)	290 (110)	273 (133)	299 (96)	0.3
**Plasma HIV-1 RNA** (log_10_ copies/ml), mean (SD)	4.8 (0.7)	5.0 (0.7)	4.7 (0.7)	0.07
**Subtype B virus**	75 (88)‡	25 (89)‡	50 (88)‡	1.0
**Initiation of cART during chronic HIV-infection**				
- triple-class therapy	6 (6)	3 (10)	3 (5)	0.4
- dual-class NNRTI	61 (65)	24 (77)	37 (59)	0.1
- dual-class PI	27 (29)	4 (13)	23 (37)	0.03

Data are n (%) unless indicated otherwise. MSM, men who have sex with men; PHI, primary HIV infection.

‡ 9 missing patients: 3 in the non-early treated and 6 in the early treated group.

**Figure 1:**
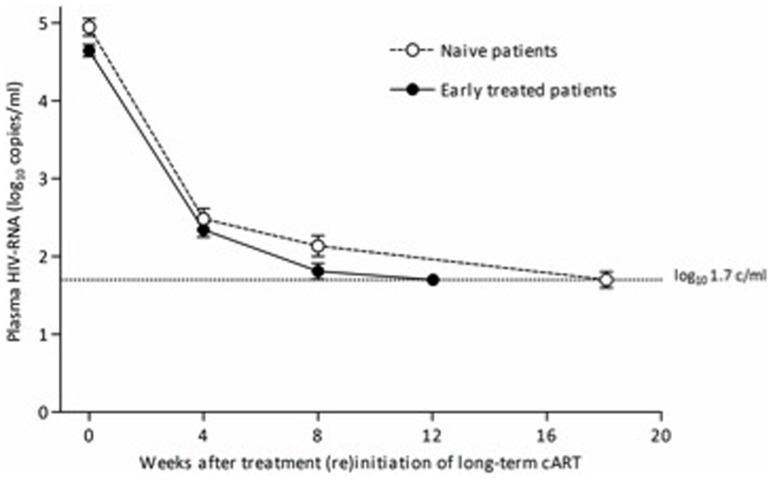
Viral decay after treatment (re)initiation of long-term cART in naive and early treated patients.

**Figure 2:**
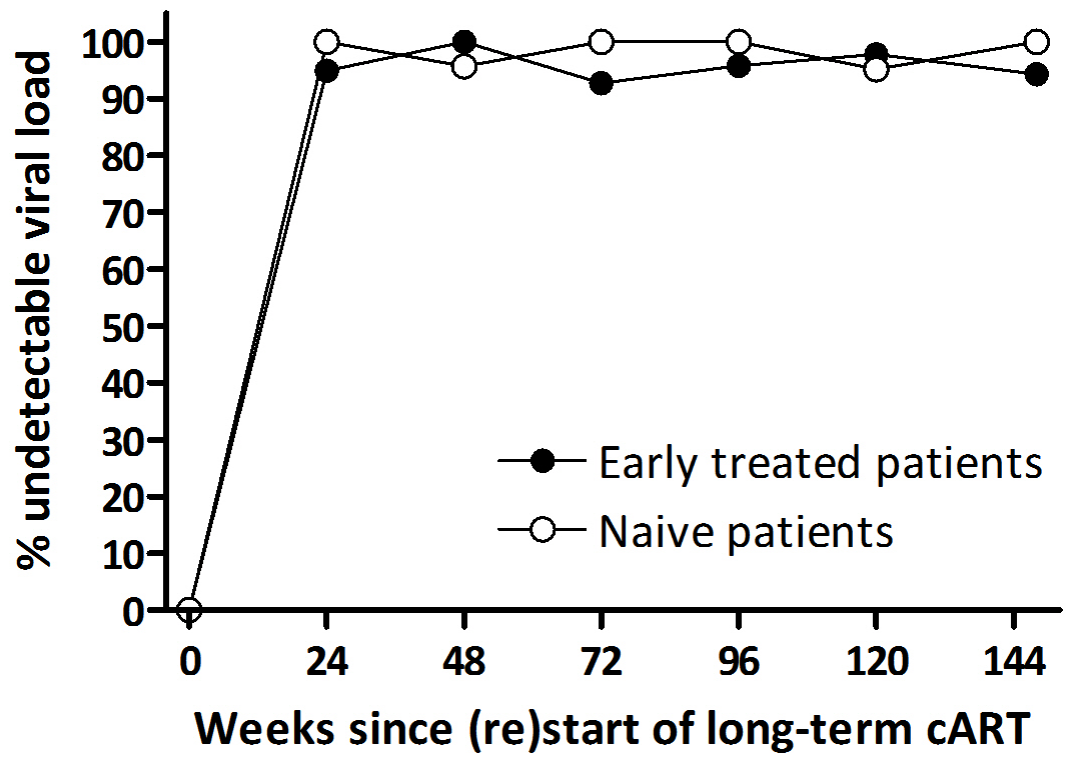
Proportion of patients with an undetectable pVL after (re)initiation of long-term cART over a period of 144 weeks in naive and early treated patients.

**Figure 3:**
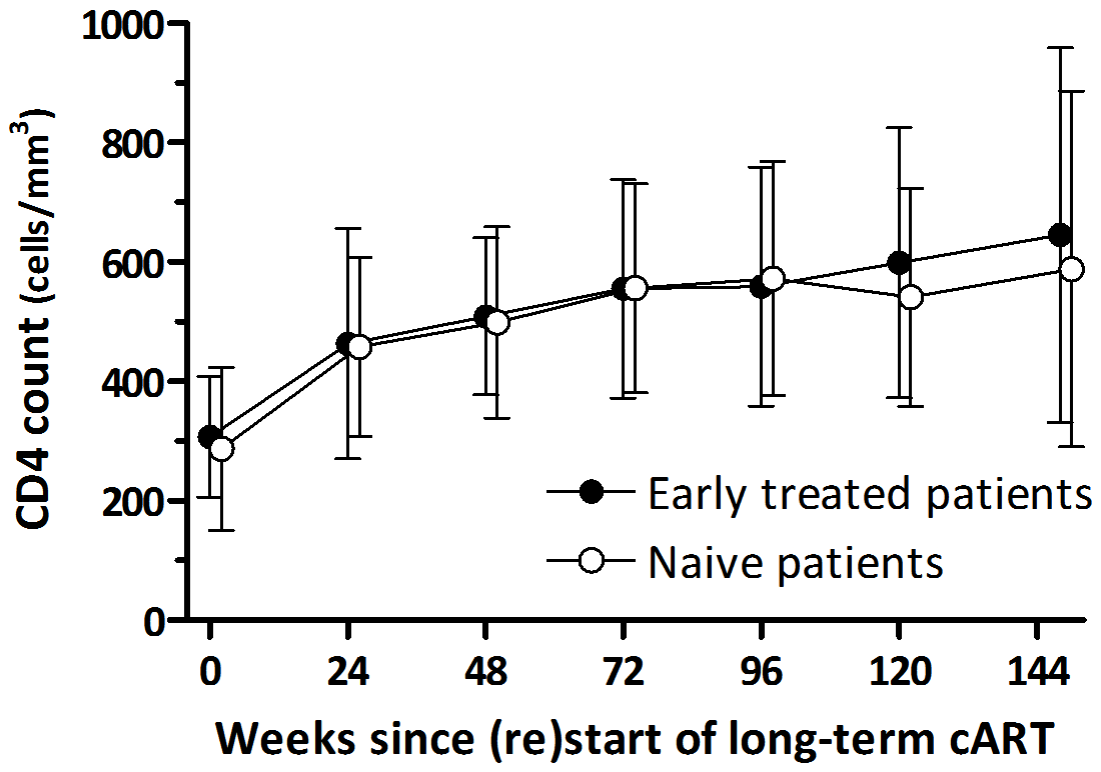
CD4 count recovery after treatment (re)initiation of long-term cART in naive and early treated patients.

All naive and early treated patients achieved viral (re)suppression. The mean number of pVL measurements after (re)start of cART, upto and including the first undetectable pVL, was 2.3 (SD 1.2) in the naive and 2.3 (1.5) in the early treated patients, respectively (*P* = 0.87). The mean interval between pVL measurements was not significantly different for the two groups (53 (SD 23) vs. 50 (20) days, respectively, *P* = 0.54). Viral decay after treatment (re)initiation was similar between the naive and early treated patients: during the first four weeks the pVL decreased with 0.62 and 0.58 log_10_ copies/ml/week respectively (*P* = 0.32), from week four to eight with 0.087 and 0.13 log_10_ copies/ml/week (*P* = 0.37), and from eight weeks onward with 0.043 and 0.027 log_10_ copies/ml/week (*P* = 0.23) (Figure 1). Adjusting the viral decay for the difference in pVL at (re)start of long-term cART also showed no significant differences between the two groups (data not shown). The median time to viral (re)suppression in naive and early treated patients was 16.4 (IQR 9.6–20.6) and 16.6 (8.7–21.0) weeks, respectively (log-rank, *P* = 0.72). Figure 2 demonstrates that three years after (re)initiation of long-term cART the proportion of patients having an undetectable pVL was not significantly different between the naive and early treated patients. In addition, the CD4 count recovery after treatment (re)initiation showed no differences over time between the two groups (Figure 3).

In the Cox analysis early treatment during PHI as compared to no treatment was not associated with time to viral resuppression (HR 0.81 (95% CI 0.27–2.39); *P* = 0.70). As expected, a higher vireamia at (re)start of long-term cART was predictive for a longer time to viral suppression (HR 0.37 per 1 log_10_ copies/ml increase (95% CI 0.25–0.56); P<0.001). Other parameters, including reinitiating with an NNRTI-based regimen or with triple-class therapy, were not associated with time to viral (re)suppression (data not shown).

**Figure 4:**
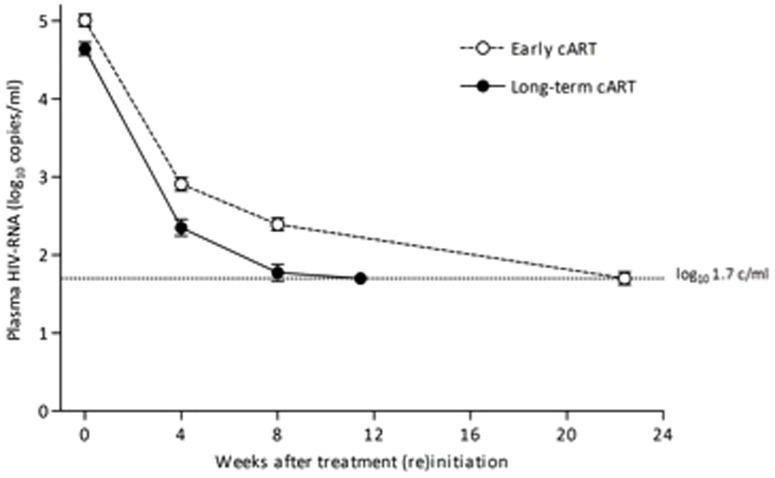
Viral decay after treatment initiation of early cART and subsequent long-term cART in the early treated patients.

In the early treated group, the median time to viral (re)suppression (Kaplan-Meier estimate) was longer in the early treatment phase compared to the phase of long-term cART: 21.7 (IQR 12.0–24.7) and 16.6 (8.7–21.0) weeks (log-rank, *P* = 0.043), respectively. This difference in time to first undetectable viral load measurement appeared to be primarily driven by the higher viral load at the time of start of early cART compared to the viral load at the restart of cART in the phase of long-term cART. Therefore, we additionally constructed mixed linear regression models comparing viral decay during the early treatment episode and subsequent restart of long-term cART (Figure 4). During the first four weeks the pVL decreased with 0.52 and 0.57 log_10_ copies/ml/week, respectively (*P* = 0.16), from week four to eight with 0.13 and 0.14 log_10_ copies/ml/week (*P* = 0.70), and from eight weeks onward with 0.048 and 0.021 log_10_ copies/ml/week (*P* = 0.003).

## Discussion

Temporary cART during PHI did not select for clinically relevant drug resistance mutations and was associated with a durable and persisting virologic response after subsequent reinitiation of long-term cART. Our data thereby support the use of early treatment during PHI. Of note, the slower decline of the pVL from 8 weeks onward in the second treatment period of the early treated patients is probably an artefact of the model, because patients had a lower baseline pVL at restart than during early treatment and their pVL was usually already undetectable by week 8.

We were not able to perform resistance testing after TI in 7/63 (11%) of the early treated patients. We therefore cannot exclude with certainty that there might have been selection of drug resistance after TI in these patients. This is in particular relevant for patients who interrupted an NNRTI-based regimen, because of the long half-life of NNRTIs. However, in all seven patients, irrespective of the regimen, the pVL was resuppressed upon restart, which virtually excludes clinically important mutations. The early treated patients reinitiated long-term cART more often with a boosted PI than naive patients. Many early treated patients preferred not to restart an NNRTI because of side-effects they had experienced previously during the early cART period, and therefore favoured a PI-containing regimen.

Our study is supported by another study in which 37 PHI-patients were treated with temporary early cART and no drug resistance was observed after TI [4]. However, in this study the NNRTI was stopped 96 h before the NRTI-backbone. Because NNRTIs have a slower metabolism and a low genetic barrier to resistance, simultaneous TI of an NNRTI-containing regimen may result in a period of NNRTI-monotherapy, which may select for drug resistance mutations [5]. NNRTI-drug resistance mutations that were selected after intrapartum exposure to single-dose nevirapine in HIV-infected women have been associated with decreased virologic response after subsequent treatment with an NNRTI-containing regimen [6], [7]. Noteworthy, the pVL in these women exposed to single-dose nevirapine was much higher than the pVL in a controlled TI-setting in which patients have an undetectable pVL at TI. In the SMART trial [8], NNRTI-drug resistance mutations were more common in case of simultaneous TI than in case of a staggered or a switched interruption, in which the NNRTI is replaced by a boosted PI [9]. However, in SMART most drug combinations included a zidovudine/lamivudine-backbone in combination with an NNRTI [8], whereas in our trial half of the patients were using a tenofovir-containing regimen, which has a longer half-life [10], and together with an NNRTI forms a more balanced regimen that is less prone to development of drug resistance when treatment is discontinued simultaneously. To date, there is no clear consensus how to stop cART regimens [11]. In our study we found no indication for selection of drug resistance mutations after interrupting all drugs simultaneously once an undetectable pVL had been reached.

In conclusion, temporary cART during PHI was not associated with a reduced virologic response after subsequent reinitiation of long-term cART. Concerns for developing drug resistance mutations after TI have not been substantiated. Therefore, fear of drug resistance development is not a valid argument to refrain from early treatment during PHI: even if patients interrupt early treatment, they still have a good and durable response after subsequent reinitiation of cART. A question is whether early cART should be interrupted at all, butin our experience some patients prefer to interrupt treatment after a period of early treatment. In brief, this study contributes to the increasing data supporting (temporary) early cART during PHI.
